# TikTok as an Educational Tool for Kidney Stone Prevention

**DOI:** 10.7759/cureus.48789

**Published:** 2023-11-14

**Authors:** Bassel Salka, Malik Aljamal, Firas Almsaddi, Hamza Kaakarli, Lauren Nesi, Kenneth Lim

**Affiliations:** 1 Urology, University of Michigan, Ann Arbor, USA; 2 Orthopaedics, Wayne State University, Detroit, USA; 3 Orthopaedics, Michigan State University, Detroit, USA; 4 Cardiology, University of Michigan, Ann Arbor, USA; 5 Urology, Detroit Medical Center, Detroit, USA

**Keywords:** tiktok, education, nephrolithiasis, prevention, urolithiasis, kidney stones

## Abstract

Introduction: The incidence of kidney stones in the United States continues to increase. Although dietary interventions have shown to be effective in reducing stone events, adherence to medical management continues to be a limiting factor. For that reason, patient education has become a focus of many physicians. TikTok, a social media application with over one billion users worldwide, has emerged as an online center for medical knowledge sharing by physicians and non-physicians alike. TikTok users share kidney stone prevention information through short informational videos directly to the general public. Little is known regarding the quality of medical advice provided in these videos. The purpose of this study was to evaluate the reach and quality of kidney stone prevention information on TikTok.

Methods: We conducted a cross-sectional analysis of renal stone prevention content on TikTok utilizing the search term #kidneystoneprevention to assess all the videos appearing on October 10th, 2022. Only videos in English, related to the topic, and with >1000 views were included. Videos were analyzed for descriptive statistics, including views, uploader profession, and stone prevention recommendations. Videos were assessed utilizing Denver International Study Center of Evaluative Rating of Information (DISCERN), a questionnaire used to appraise the quality of consumer health information (maximum score of 80 per video). The one-way analysis of variance (ANOVA) was used to determine statistical significance groups.

Results: Out of a total of 131 videos, 87 fit the inclusion criteria, resulting in a total of 8.75 million views. An average DISCERN score of 27.0 was observed. Only eight videos were published by physicians, of which the average DISCERN score of 35.3 was significantly greater than an average score of 26.2 for non-physicians (p<0.05). The most common recommendation was increased fluid intake (38.0%) followed by monitoring calcium levels (9.02%) and decreasing oxalate-rich foods (9.2%).

Conclusions: Kidney stone prevention content on TikTok has a wide reach with millions of consumers. The majority of videos fail to match American Urological Association recommendations regarding diet therapies for stone prevention. Further research is needed to understand the extent of kidney stone prevention misinformation on social media and how it contributes to patient outcomes. Increased engagement in TikTok by urologists and health organizations may improve public education.

## Introduction

The prevalence of kidney stones in the United States has increased to about 11% [1] despite strongly supported kidney stone prevention practices [2,3]. Although dietary interventions described in the American Urologic Association (AUA) guidelines [4] like increasing fluid, controlling calcium, and decreasing oxalate have shown efficacious in reducing stone events, adherence to medical management continues to be a limiting factor. As a result, many urologists have focused on improving patient education on medical management to prevent stone formation. With the demonstration of social media as a popular and effective means of disseminating health information [5-7], many physicians have turned to social media for stone prevention education [8]. With over one billion users worldwide, TikTok is the fastest-growing social media platform in the world [9] and has become a means for dissemination of all types of information, including healthcare content. 

Little is known about the quality or quantity of kidney stone prevention information on TikTok. With kidney stone prevention information on the internet historically deemed incomplete [10], TikTok may serve as a positive catalyst for positive patient education given its attractive short-video style and editing software. Conversely, it may also serve as a form for mass distribution of incomplete or inaccurate practices for stone prevention. For example, a study published in 2022 comparing the quality of information about urinary tract infections between YouTube and TikTok found that YouTube videos had higher median scores for scientific information, credibility, and less misinformation compared to TikTok [11].

The purpose of this study was to evaluate the quality of kidney stone prevention information on TikTok using the Denver International Study Center of Evaluative Rating of Information (DISCERN) media evaluation criteria. DISCERN is a standardized questionnaire used to appraise consumer health information online. The study consisted of three objectives: 1) to evaluate the quality of kidney stone prevention content on TikTok, 2) to compare video quality based on creator characteristics and video content, and 3) to examine how existing TikTok videos match the latest AUA recommendations. This study can illuminate gaps in stone prevention information on TikTok and identify opportunities for kidney stone prevention education and distribution.

## Materials and methods

Video selection

**Figure 1:**
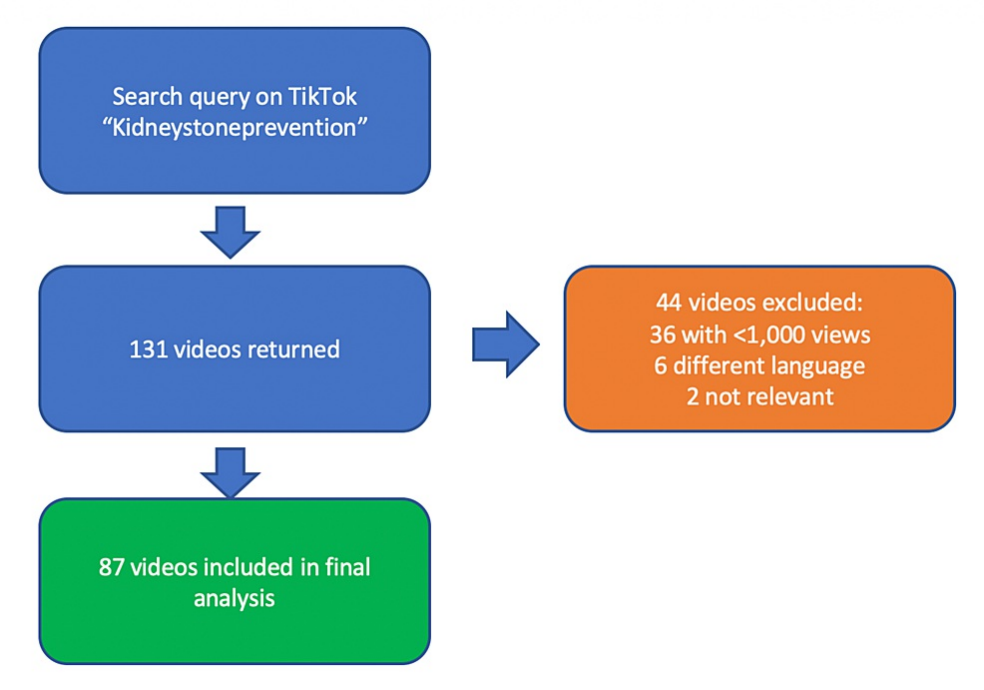
Video Inclusion/Exclusion Flow Diagram

The cross-sectional analysis of kidney stone prevention content on TikTok was performed by creating a new account and entering the search term #kidneystoneprevention. The search returned a total of 131 videos on October 10th, 2022. All videos in English and with more than 1,000 views were included. Videos that did not relate to the topic of kidney stones were excluded. Of the 131 videos initially returned by the search, 6 were not in English, 36 had fewer than 1,000 views, and 2 did not mention kidney stones. The remaining 87 videos were used for content analysis (Figure 1).

Data collection

Two reviewers separately recorded information on each video’s creator, popularity, DISCERN score, and video content. When the two reviewers disagreed on a particular piece of data, a third reviewer reviewed the video and settled the difference. Inter-reliability was ensured by confirming a minimum of 90% agreement across all reviewers.

Identification of the content creator as a physician was ascertained through self-identification in the video itself or through the creator’s homepage. Whenever this information was not provided, the user was designated as a non-physician. Videos were categorized as either “Management”, “Educational”, “Advertisement”, or “Anecdotal” types of videos following the practice of other studies on the same topic [12]. Popularity of a video was measured as the number of views, number of likes, and number of comments in each video. Lastly, each reviewer recorded a list of kidney stone prevention recommendations each video mentions.

The DISCERN evaluation system was used to assess the quality of each video. The system consists of a 16-question survey that takes the sum of all questions answered with the Likert scale (“5” a strong yes and “1” a strong no). Although no consensus to a “good” or “bad” DISCERN score has been established as far as we know, the standardized methodology for calculating a video’s total score provides an effective tool for comparing video quality relative to one another.

Data analysis

Video information was categorized into various creator groups and types of content. A one-way variance analysis (ANOVA) was used to determine statistically significant differences in DISCERN scores between these groups. The proportion of videos that mention each AUA recommendation was determined by evaluating every video’s content.

## Results

Overall video characteristics

A total of 87 videos met the inclusion criteria. Physicians accounted for 9% of all content creators. Males accounted for 25% while females accounted for 44% (21% unknown). The most common video type was “Management” accounting for 49% of all videos. The least common types of videos were advertisements and anecdotal videos at 6%. However, advertisements were the most popular type of video accounting for the most views, likes, and comments. The average DISCERN score across all videos was 27.0. Table 1 lists the popularity and DISCERN score for each of these subgroups.

**Table 1:** Overview of Video Characteristics

	Number of Videos (%)	Mean No. Views	Mean No. Likes	Mean No. Comments	Mean Discern Scores
Content Creator					
Physician	8 (9)	71,620	592	54	35.25
Non-Physician	79 (91)	104,825	989	61	26.19
Sex:					
Male	22 (25)	41,058	556	28	35.71
Female	44 (51)	153,839	1235	91	24.73
N/A	21 (24)	40,442	777	21	22.71
Video Type:					
Management	43 (49)	66,435	1111	44	30.81
Educational	14 (16)	8,462	152	6	29.43
Advertisement	6 (7)	333,622	559	314	24.16
Anecdotal	6 (7)	6,363	277	12	23.66
Other	18 (21)	207,924	1608	76	18.16

Physician vs non-physician

Videos made by physicians had an average DISCERN score of 35.3, significantly greater than an average score of 26.2 for non-physicians (p<0.05). However, they averaged 31.7% less views and 40.1% less likes.

Top 10 most viewed videos

No statistically significant difference exists between the DISCERN scores of the most viewed videos and the remaining 77.

Video type

Advertisements about kidney stone-related products were the most viewed and commented video type with an average of 333,622 views and 314 comments per video. No advertisements reported on a product discussed in PubMed, Cochrane, or Medline. The average DISCERN score for advertisements (24.16) was lower than the average of all videos (27.02). 

TikTok recommendations

The only AUA stone evaluation guidelines acknowledged were urinalysis (n=4, 4.6%) and 24-hour urine collection (1.1%, n=1). The most common AUA diet therapy recommendations mentioned in the videos were increased fluid intake (n=33, 38.0%) followed by monitoring calcium levels (n=8, 9.02%) and decreasing consumption of oxalate-rich foods (n=8, 9.2%). The most commonly advised nutritional supplementation was lemon water (n=7, 8.0%). No pharmacologic therapies were addressed in any of the videos. A total of 11.5% (n=10) of videos did not mention any AUA evaluation or dietary therapy elements and 28.7% (n=25) reported only one element. A breakdown of the number of videos that acknowledge AUA diet therapy recommendations is presented in Table 2. No significant differences were found among the DISCERN scores of videos that mentioned AUA stone evaluation and diet therapy guidelines. 88% (n=7) of physicians' recommendations were in line with AUA. Additionally, 23 of the top 25 most viewed videos were in line with AUA recommendations.

**Table 2:** AUA Medical Management Guidelines and Video Content

AUA Recommendation	Number of Videos	Avg. DISCERN Score	Proportion of Videos
Increase fluid intake	28	27.02	32.2%
Limit sodium	4	27.02	4.6%
Control calcium (1,000-1,200mg)	8	29.21	9.2%
Limit oxalate	8	29.07	9.2%
Increase fruit and vegetables	6	26.25	6.9%
Limit non-dairy protein	4	27.02	4.6%

## Discussion

Out of a total of 131 videos, 87 fit the inclusion criteria, resulting in a large total viewership of 8.75 million. An average DISCERN score of 27 (maximum score of 80) was observed. Only eight videos were published by physicians, of which the average DISCERN score was significantly greater than the average score for non-physicians. This finding of low physician engagement on TikTok despite statistically higher video quality by physician creators suggests that urologists should engage more on TikTok.

Many studies evaluating urology content on TikTok have reported similar findings. For example, one study evaluating men’s health on TikTok revealed that only 10.3% of all content creators were physicians and that physician videos were more accurate than non-physicians. Similarly, a study examining urinary tract infection information on TikTok in 2022 similarly found that only 20% of the creators were physicians [11]. Both these studies concluded that although TikTok was a more popular social media platform than Instagram and YouTube, the content of healthcare information on TikTok was statistically less accurate. Similar conclusions have been reached on evaluations of TikTok content quality on varicoceles [13] and prostate cancer [14].

The observation that physicians on TikTok create higher quality videos is of no surprise. A survey study assessing patient satisfaction with kidney stone information advice during admission for acute renal colic concluded that “traditional patient education regarding kidney stones can be further strengthened through the use of a concise, informative, and readily accessible patient education video during and after point of care” [15]. TikTok videos provide urologists the opportunity to provide information on this description exceptionally well. For this reason, the evaluation of information distribution on TikTok has also gained popularity in fields outside of urology like radiology [16] and ophthalmology [12].

Encouragingly, the possibility of significantly increasing urologist engagement on TikTok with associated high quality has been demonstrated in other parts of the world. A study of the keyword “diabetes” in Chinese in 2021 included a total of 199 videos and found that health professionals contributed to 69.3% of all videos and that the DISCERN scores across these videos were higher than general users [17]. A similar finding was found in a Chinese analysis of TikTok for information on anal fissures in which 93.4% of all videos were created by physicians [18].

Increased engagement of urologists on TikTok is highlighted by the potential the platform has to reach incredibly large populations that traditional education fails to reach. The aforementioned study evaluating men’s health content on TikTok recorded about 2.3 billion total views across 234 videos (average ~9.8 million views/video) with the total number of views about testosterone, vasectomies, and erectile dysfunction at 703.5 million, 318.8 million, and 42.2 million respectively [19]. Large counts of viewership have also been recorded outside urology; a cross-sectional study of 101 videos discussing dry eyes resulted in a total of 98,170,746 views (average ~ 1.0 million views/video) [12]. Although the reason for the relatively low number of views of kidney stone prevention content is unclear, the tremendous viewership of other health conditions that may be targeting similar patient demographics reveals an enormous market for patient education. TikTok users span all ages, nationalities, and genders across the world [9]. As a result, the platform is well positioned to provide dietary counseling for patients before they ever have a stone event. This low-cost means of reaching a large audience has driven the increasingly popular study of social media for medical education within urology [20].

Although this study is the first to evaluate the kidney stone prevention information on TikTok, some limitations must be addressed. First, there is potential for incorrect identification of the physician identification of the content creator as this was ascertained on the basis of the information provided in the user's profile or self-identified in the video. Whenever this information could not be obtained, the user was designated as a non-physician. Second, we limited our search term to #kidneystoneprevention without using any other search iterations. Although this can result in an incomplete sample of all kidney stone prevention information on TikTok, our practice of limiting the search to just one term is in line with similar TikTok studies in other fields [12,18]. Third, we limited our search findings to English when stone prevention information may be more robust in other languages. According to the U.S Census Bureau, 21.6% of people in the U.S speak a language other than English at home [21]. Lastly, this was a cross-sectional study based upon the most popular videos and posts at a single point in time. Given the nature of TikTok, the most popular videos and posts constantly evolve over time and the reported popularity statistics will change over time.

Limitations notwithstanding, the findings of this study are significant for a few reasons. First, the finding that kidney stone prevention content on TikTok has a reach of millions of consumers highlights the reach of the platform. However, the popularity of the kidney stone prevention videos is small in comparison to other fields in urology like men’s health and UTI prevention, indicating a need for more content. Second, our study identified that the majority of videos failed to match AUA recommendations regarding diet therapies for stone prevention. Our findings suggest that this may be in part due to the low number of videos that are created by physicians who on average produce significantly higher quality videos. When comparing our study to similar TikTok studies in other healthcare subjects, the pattern of improved video quality with increased physician participation in content creation is seemingly ubiquitous. This suggests that increased engagement in TikTok by urologists and health organizations may improve public education. Lastly, our study identified specific areas of misinformation on TikTok which can serve as targets for further targeted kidney stone prevention education.

## Conclusions

Kidney stone prevention content on TikTok has a wide reach with millions of consumers. The majority of videos fail to match AUA recommendations regarding diet therapies for stone prevention. Increased engagement in TikTok by urologists and health organizations may improve public education.
